# Supplementary material for: Individual retrotransposon integrants are differentially controlled by KZFP/KAP1-dependent histone methylation, DNA methylation and TET-mediated hydroxymethylation in naïve embryonic stem cells
Source: Epigenetics Chromatin. 2018 Feb 26;11:7. doi: 10.1186/s13072-018-0177-1 (PMC6389204; doi:10.1186/s13072-018-0177-1)
Supplement: Supplementary file 11 — Additional file 11. Pattern analysis. [file 13072_2018_177_MOESM11_ESM.zip › Patterns analysis/DataTables/extensions/FixedHeader/examples/index.html]

FixedHeader examples - FixedHeader examples


# FixedHeader example FixedHeader examples

At times it can be useful to ensure that column titles will remain always visible on a table, even
when a user scrolls down a table. The FixedHeader plug-in for DataTables will float the `thead` element above the table at all times to help address this issue.
The column titles also remain click-able to perform sorting. Key features include:

- Fix the header to the top of the window
- Ability to fix the footer and left / right columns as well
- z-Index ordering options

### Examples

- Basic initialisation
- Header and footer fixed
- Header, left and right all fixed
- Multiple tables
- z-index order control

Please refer to the DataTables documentation for full
information about its API properties and methods.  
Additionally, there are a wide range of extras and
plug-ins which extend the capabilities of
DataTables.

DataTables designed and created by SpryMedia Ltd © 2007-2014  
DataTables is licensed under the MIT license.
